# Readability of extraspinal organs on scout images of lumbar spine MRI according to different protocols

**DOI:** 10.1371/journal.pone.0251310

**Published:** 2021-05-13

**Authors:** Ja Yeon You, Joon Woo Lee, Jiwoon Seo, Jee Won Chai, Hee Dong Chae, Heung Sik Kang

**Affiliations:** 1 Department of Radiology, S & K Hospital, Daejeon, Korea; 2 Department of Radiology, Seoul National University Bundang Hospital, Seongnam-si, Gyeonggi-do, Korea; 3 Department of Radiology, Seoul National University College of Medicine, Seoul, Korea; 4 Department of Radiology, Seoul Metropolitan Government—Seoul National University Boramae Medical Center, Seoul, Korea; 5 Department of Radiology, Seoul National University Hospital, Seoul, Korea; Johns Hopkins School of Medicine, UNITED STATES

## Abstract

**Background:**

Scout images of lumbar spine MRI often include the extraspinal organs, which are barely included in routine MRI and can be a potential cause of lumbar pain.

**Purpose:**

To evaluate the readability of scout images for extraspinal organs in lumbar spine MRI according to different protocols.

**Materials and methods:**

A total of 150 patients who underwent 1.5 T or 3 T lumbar spine MRI from March to September 2015 at three hospitals with different scout image protocols, were selected. Two radiologists independently reviewed the scout images to investigate whether exclusive diagnosis of major diseases involving the femoral head, femoral neck, sacroiliac joint, and kidneys was possible. Readability levels were divided into four categories: definitely, possibly, limited, and non-evaluable. The readability of scout images according to the protocols was compared using Chi-square test. Interobserver agreement for the readability level of scout images was assessed using weighted κ statistics.

**Results:**

Of 150 patients, “definitely evaluable” cases classified by two readers were 50–62 (33.3–41.3%) for femoral head (κ = 0.63–0.71), 37–66 (24.7–44.0%) for femoral neck (κ = 0.41–0.48), 72–93 (48.0–62.0%) for sacroiliac joint (κ = 0.35–0.37), and 63–73 (42.0–48.7%) for kidneys (κ = 0.45–0.47). More than 50% of femoral heads were classified as readable (definitely or possible evaluable) cases by two readers with excellent interobserver agreement. The readability level of scout images was significantly different according to image protocols including the MRI sequence, number of coronal plane slices, and intersection gap of coronal plane slices (*p*≤0.015).

**Conclusion:**

Scout images of lumbar spine MRI may be readable enough to rule out some major diseases of extraspinal organs. Standardization of the protocol will be needed to validate the potential role of scout images for screening extraspinal organs.

## Introduction

Scout images are referred using various terminologies including scanogram, surview, topogram, localizer image, or pilot scan. Scout images are critical for MRI as guiding images that aid in the determination of appropriate scan range, levels, and angles. They are obtained rapidly in approximately a minute and comprise low spatial resolution images. Most radiologists and physicians rarely read scout images in routine practice due to time constraints and indifferences. Scout images are not even uploaded to the Picture Archiving and Communication System (PACS) in some hospitals. However, the quality of scout images has continued to improve along with the evolution and innovation of MRI technology. Several previous studies have documented incidental findings on MRI scout images of various organs [1–17]. Although most incidental findings are benign and asymptomatic, some may be related to clinically significant diseases including malignant tumors [3, 4, 6, 9–16]. However, the image sequence, composition, and quality of scout images differ depending on the manufacturer or institutions because they are obtained at the discretion of technologists or with the default setting of MRI without a standard protocol. Therefore, the clinical efficacy of scout images in daily practice remains debatable.

Scout images of lumbar spine MRI are usually obtained in coronal, axial, and sagittal planes of large field of view (FOV) encompassing the lower thoracic spine to the coccyx as a center point of the iliac crest; however, no standard protocols exist. Therefore, scout images of lumbar spine MRI often include the femoral head, femoral neck, sacroiliac joint, and kidneys, which are barely included in routine spine MRI and may be a potential cause of lumbar pain [1, 3, 13, 16]. In routine practice, radiologists at the authors’ institutes sometimes review scout images to find a clue for major diseases of the extraspinal organs that require differential diagnosis due to similar symptoms, especially when there is no lesion or when symptoms are not well explained on the standard lumbar spine MRI. However, no detailed studies on the potential usefulness of scout images for extraspinal organs in lumbar spine MRI have been performed.

Thus, the aim of this study was to evaluate the readability of scout images for extraspinal organs in lumbar spine MRI according to different protocols.

## Materials and methods

### Ethics approval

This retrospective study was approved by the Institutional Review Boards from Seoul Metropolitan Government—Seoul National University Boramae Medical Center (IRB No: 20190704/10-2019-55/081), Seoul National University Hospital (IRB No: J-1609-018-789), and Seoul National University Bundang Hospital (IRB No: B-1906/546-106). This study was conducted in accordance with relevant guidelines and regulations and followed the tenets of the Declaration of Helsinki. Requirement of informed consent from participants was waived by the Institutional Review Boards of each hospital due to the retrospective study design.

### Study population

**Fig 1:**
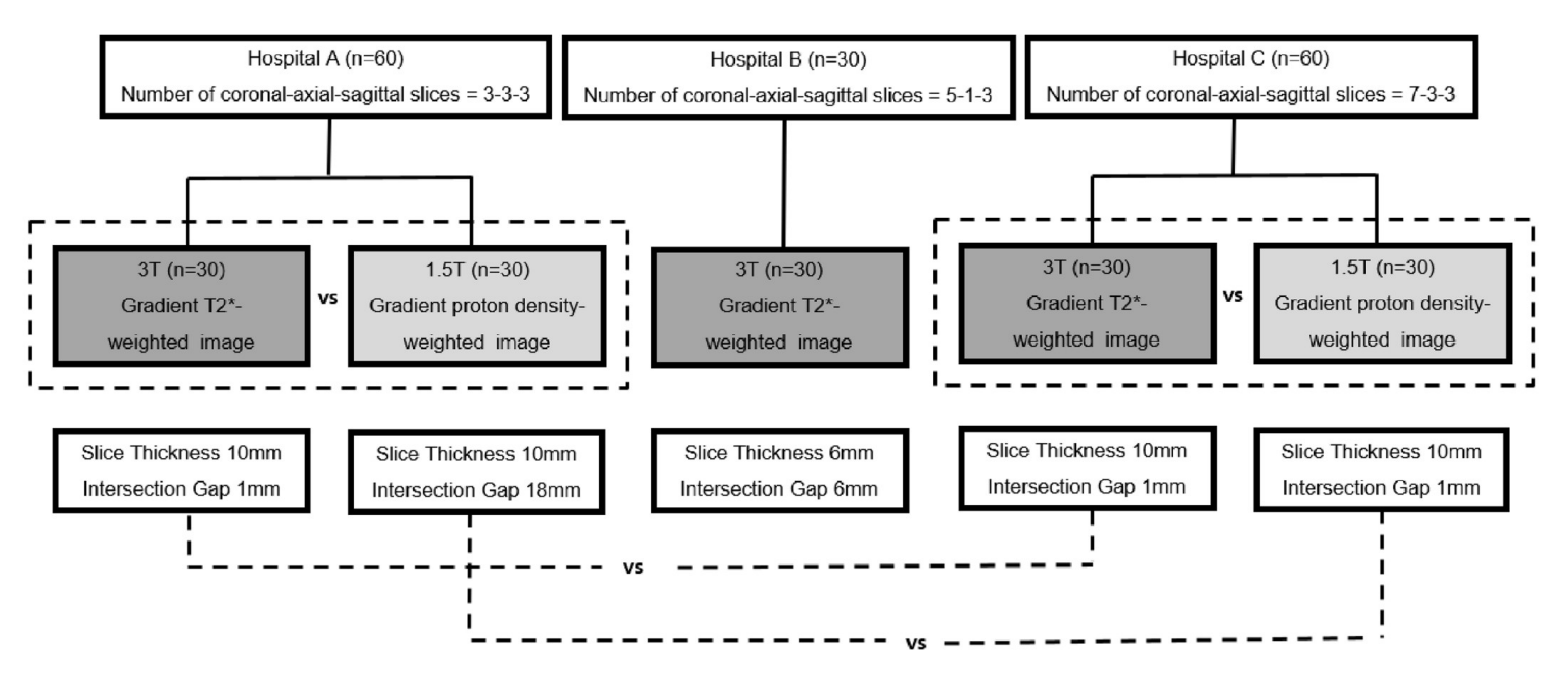
Flow chart of the study design.

We collected data of 153 patients who underwent 1.5 T or 3 T lumbar spine MRI consecutively from March to September 2015 at three different hospitals (hospital A, B, and C) with different scout image protocols. Immediate postoperative lumbar spine MRI was excluded from data collection due to its different MRI test name. Three patients were excluded due to whole spine scanning (n = 1), no uploaded scout image (n = 1), and incomplete scout image (n = 1) uploaded on the PACS from consecutive data collection. A total of 150 patients were included. Of these, 90 patients comprising 30 patients each in hospitals A, B, and C underwent 3 T MRI, and the remaining 60 patients comprising 30 patients each in hospitals A and C underwent 1.5 T MRI (Fig 1).

### MR imaging protocol

Lumbar spine MRIs in all the hospitals were performed with the patient in the supine position. Scout images consisted of 3-3-3 slices each of coronal, axial, and sagittal planes in hospital A; 5-1-3 slices in hospital B; and 7-3-3 slices in hospital C. At hospital A, lumbar spine MRI was performed on 1.5 T (Achieva, Philips Medical Systems, Best, the Netherlands) or 3 T (Achieva, Philips Medical Systems Best, the Netherlands) scanners. Coronal, axial, and sagittal scans with gradient echo sequence were acquired on 1.5 T (TR/TE, 7.5 or 22.3 ms/3.8 ms; flip angle, 45°; slice thickness, 10 mm; FOV, 400×400 mm; matrix, 256×192; number of excitations, 2; intersection gap in the coronal plane, 18 mm, acquisition time of 25 sec) and 3 T MRIs (TR/TE, 10.4 ms/6.9 ms; flip angle, 20°; slice thickness, 10 mm; FOV, 450×450 mm; matrix, 288×216; number of excitations, 2; intersection gap in the coronal plane, 1 mm, acquisition time of 41 sec). At hospital B, lumbar spine MRI of all patients was performed on 3 T (Magnetom Verio, Siemens Medical Solutions, Erlangen, Germany) scanners. Coronal, axial, and sagittal scans with gradient echo sequence (TR/TE, 6.5 ms/2.9 ms; flip angle, 20°; slice thickness, 6 mm; FOV, 380×380 mm; matrix, 256×205; number of excitations, 1; intersection gap in the coronal plane, 6 mm, acquisition time of 16 sec) were acquired. At hospital C, lumbar spine MRI was performed on 1.5 T (Gyroscan Intera, Philips Medical Systems, Best, the Netherlands) and 3 T (Achieva, Philips Medical Systems, Best, the Netherlands) scanners. Coronal, axial, and sagittal scans with gradient echo sequence were acquired on 1.5 T (TR/TE, 51.83–51.86 ms/3.75 ms; flip angle, 45°; slice thickness, 10 mm; FOV, 400×400 mm; matrix, 256×192; number of excitations, 2; intersection gap in the coronal plane, 1 mm, acquisition time of 60 sec) and 3 T MRIs (TR/TE, 10.34 ms/6.91 ms; flip angle, 20°; slice thickness, 10 mm; FOV, 500×500 mm; matrix, 332×128; number of excitations, 1; intersection gap in the coronal plane, 1 mm, acquisition time of 51 sec).

### Image analysis

Scout images were saved as anonymized JPEG files and independently reviewed by two radiologists specialized in musculoskeletal imaging with >6 years of experience in spine MRI. The radiologists provided subjective assessment of the readability of scout images indicating whether exclusive diagnosis of major diseases is possible in the femoral head, femoral neck, sacroiliac joint, and kidneys. Major diseases of each organ considered while reviewing the images were limited to avascular necrosis of the femoral head, femoral neck fracture, sacroiliitis, and hydronephrosis suspected to be related to vague low back complaints. For bilateral organs, the left and right sides were separately analyzed. Readability levels were divided into four categories: definitely, possibly, limited, and non-evaluable. “Definitely evaluable” was assigned when exclusive diagnosis was made with high probability without any doubt. “Possibly evaluable” was assigned when exclusive diagnosis was made with moderate probability owing to suboptimal image quality or minimal artifacts. “Limited evaluable” was assigned if the reader had low confidence level for exclusive diagnosis owing to incomplete coverage or pronounced artifacts. “Non-evaluable” was assigned if the reader had complete uncertainty for exclusive diagnosis owing to no coverage, poor image quality, or severe artifacts. For comparison analysis, definitely and possibly evaluable cases were assigned a label of “readable case”, while limited and non-evaluable cases were assigned a label of “non-readable case”. If scout images were designated “non-readable cases”, each radiologist commented on the cause of inability to interpret images, such as incomplete coverage, susceptibility artifacts, motion artifacts, or poor image quality.

### Statistical analyses

Statistical analyses were performed using SPSS software version 21.0 (SPSS, ‎International Business Machines Corporation, Chicago, Illinois, USA). Results are presented as frequency and percentage. Interobserver agreement for the readability level of scout images was assessed using weighted κ statistics: poor (<0.20), fair (0.21–0.40), moderate (0.41–0.60), good (0.61–0.80), and excellent (0.81–1.00) agreement [18]. Pearson’s Chi-square test and Fisher’s exact test were performed to compare the readability levels according to different scout image protocols including the MRI sequence, number of coronal plane slices, and intersection gap of coronal plane slices. The proportions of readable cases were compared between 3 T gradient T2*-weighted image (GRE T2*WI) and 1.5 T gradient proton density-weighted image (GRE PDWI) for hospitals A and C, between hospital A (three coronal plane slices) and hospital C (seven coronal plane slices) with the same slice thickness and intersection gap on 3 T GRE T2*WI, and between hospital A (three coronal plane slices with 18-mm intersection gap) and hospital C (seven coronal plane slices with 1-mm intersection gap) on 1.5 T GRE PDWI with the same slice thickness. P-values < 0.05 were considered statistically significant.

## Results

**Fig 2:**
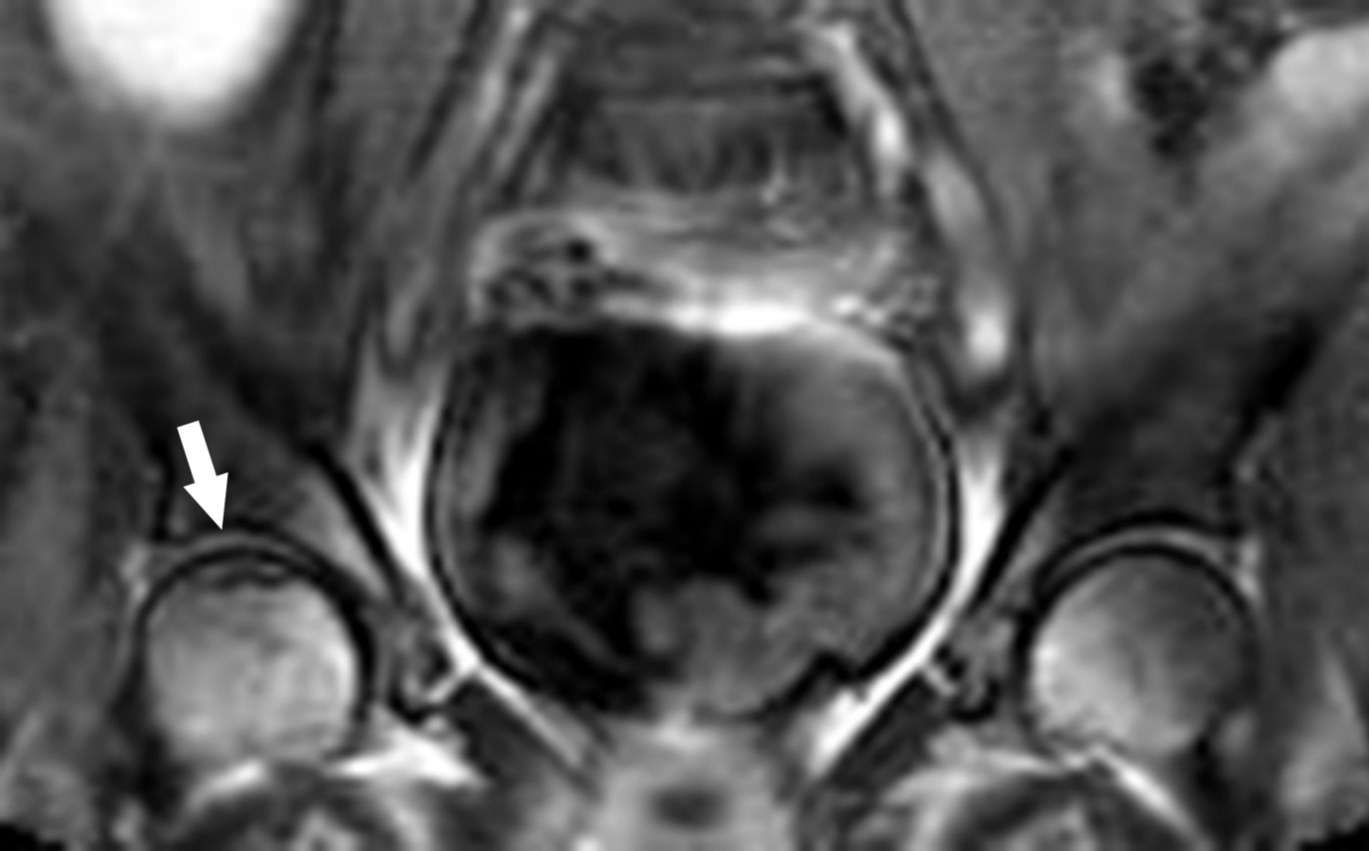
Avascular necrosis of the femoral head on the scout image. Early-stage of avascular necrosis (arrow) of the right femoral head, which is not included in the routine lumbar spine MRI, is incidentally detected on the coronal plane of the scout image.

In total, 150 patients (M:F = 72:78; mean age: 59.4 ± 16.9 years; range: 18–86 years) were included in this study. Of these, 50–62 (33.3–41.3%) for both femoral heads, 37–66 (24.7–44.0%) for both femoral necks, 72–93 (48.0–62.0%) for sacroiliac joints, and 63–73 (42.0–48.7%) for kidneys were classified as definitely evaluable cases. The number of possibly evaluable cases was 18–29 (12.0–19.3%) cases for both femoral heads, 15–30 (10.0–20.0%) cases for both femoral necks, 38–57 (25.3–38.0%) cases for sacroiliac joints, and 27–44 (18.0–29.3%) cases for kidneys (Table 1). The interobserver agreements for the readability levels of scout images were good for both femoral heads (κ = 0.63–0.71), moderate for both femoral necks (κ = 0.41–0.48) and kidneys (κ = 0.45–0.47), and fair for both sacroiliac joints (κ = 0.35–0.37). More than 50% of femoral head, sacroiliac joint, and kidney cases evaluated by reader 1 and more than 50% of all organ cases evaluated by reader 2 were classified as readable cases. For readable cases, interobserver agreements of the two readers were excellent for both femoral heads (κ = 0.92–0.93), good for both femoral necks (κ = 0.74–0.75), and moderate for sacroiliac joints (κ = 0.46–0.54) and kidneys (κ = 0.55–0.59). One case of avascular necrosis of the femoral head was incidentally detected on the coronal plane of the scout image with a prevalence of 0.67% (1/150) (Fig 2). The prevalence was higher (1.19–1.28%) when the prevalence was calculated in the readable cases of the femoral head, because some scout images did not include the femoral head or could not be evaluated due to artifacts or limited image quality. There were no other incidental findings of femoral neck fracture, sacroiliitis, and hydronephrosis.

**Table 1:** Readability of scout images for extraspinal organs on the lumbar spine MRI.

	Reader 1				Reader 2			
	Definitely evaluable	Possibly evaluable	Limited evaluable	Non-evaluable	Definitely evaluable	Possibly evaluable	Limited evaluable	Non-evaluable
	n (%)	n (%)	n (%)	n (%)	n (%)	n (%)	n (%)	n (%)
Femoral head (Rt)	50 (33.3)	29 (19.3)	40 (26.7)	31 (20.7)	53 (35.3)	25 (16.7)	42 (28.0)	30 (20.0)
Femoral head (Lt)	60 (40.0)	24 (16.0)	41 (27.3)	25 (16.7)	62 (41.3)	18 (12.0)	40 (26.7)	30 (20.0)
Femoral neck (Rt)	37 (24.7)	30 (20.0)	20 (13.3)	63 (42.0)	66 (44.0)	15 (10.0)	27 (18.0)	42 (28.0)
Femoral neck (Lt)	42 (28.0)	28 (18.7)	20 (13.3)	60 (40.0)	63 (42.0)	22 (14.7)	22 (14.7)	43 (28.7)
Sacroiliac joint (Rt)	76 (50.7)	52 (34.7)	21 (14.0)	1 (0.7)	86 (57.3)	38 (25.3)	22 (14.7)	4 (2.7)
Sacroiliac joint (Lt)	72 (48.0)	57 (38.0)	20 (13.3)	1 (0.7)	93 (62.0)	38 (25.3)	14 (9.3)	5 (3.3)
Kidney (Rt)	72 (48.0)	40 (26.7)	26 (17.3)	12 (8.0)	63 (42.0)	27 (18.0)	35 (23.3)	25 (16.7)
Kidney (Lt)	73 (48.7)	44 (29.3)	22 (14.7)	11 (7.3)	71 (47.3)	28 (18.7)	29 (19.3)	22 (14.7)

Among non-readable cases, the most common factor impeding image interpretation of both femoral heads and necks was incomplete coverage with 54.2–68.2% in the femoral head and 82.6–89.2% in the femoral neck. In sacroiliac joint, readers had difficulty reading scout images due to limited image quality and incomplete coverage (47.6–57.9% and 36.8–52.4%, respectively). In kidneys, motion artifact was the most common factor affecting image interpretation (76.7–84.8%).

A subgroup analysis by hospital revealed that more than 50% of almost all organs except for the right kidney in hospital C, which had scout image protocols with the largest number of coronal image slices, were designated as readable cases by two readers (Table 2). Among them, the proportions of readable cases in both femoral heads and necks were more than 70%. More than 50% of femoral necks were assigned as readable cases in only hospital C. Although hospital A had scout image protocols with the least number of coronal image slices, more than 50% of both femoral heads, sacroiliac joints, and kidneys were assigned as readable cases. In hospital B, less than 20% of both femoral heads and necks were assigned as readable cases, while more than 90% of both sacroiliac joints and kidneys were designated as readable cases by two readers.

**Table 2:** Comparison of the readability of scout images according to the protocols.

		Hospital A (n = 60)	Hospital B (n = 30)	Hospital C (n = 60)
		Readable case number (%)		
Femoral head (Rt)	R1	31 (51.7)	6 (20.0)	42 (70.0)
	R2	30 (50.0)	4 (13.3)	44 (73.3)
Femoral head (Lt)	R1	33 (55.0)	4 (13.3)	47 (78.3)
	R2	32 (53.3)	3 (10.0)	45 (75.0)
Femoral neck (Rt)	R1	16 (26.7)	5 (16.7)	46 (76.7)
	R2	23 (38.3)	6 (20.0)	52 (86.7)
Femoral neck (Lt)	R1	19 (31.7)	5 (16.7)	46 (76.7)
	R2	25 (41.7)	6 (20.0)	54 (90.0)
Sacroiliac joint (Rt)	R1	50 (83.3)	30 (100.0)	48 (80.0)
	R2	44 (73.3)	29 (96.7)	51 (85.0)
Sacroiliac joint (Lt)	R1	53 (88.3)	29 (96.7)	47 (78.3)
	R2	48 (80.0)	29 (96.7)	54 (90.0)
Kidney (Rt)	R1	48 (80.0)	29 (96.7)	35 (58.3)
	R2	35 (58.3)	28 (93.3)	27 (45.0)
Kidney (Lt)	R1	49 (81.7)	29 (96.7)	39 (65.0)
	R2	38 (63.3)	29 (96.7)	32 (53.3)

Scout images consisted of 3-3-3 slices each of coronal, axial, and sagittal planes in hospital A, 5-1-3 in hospital B, and 7-3-3 in hospital C; Readable case, definitely and possibly evaluable case; R, reader

**Fig 3:**
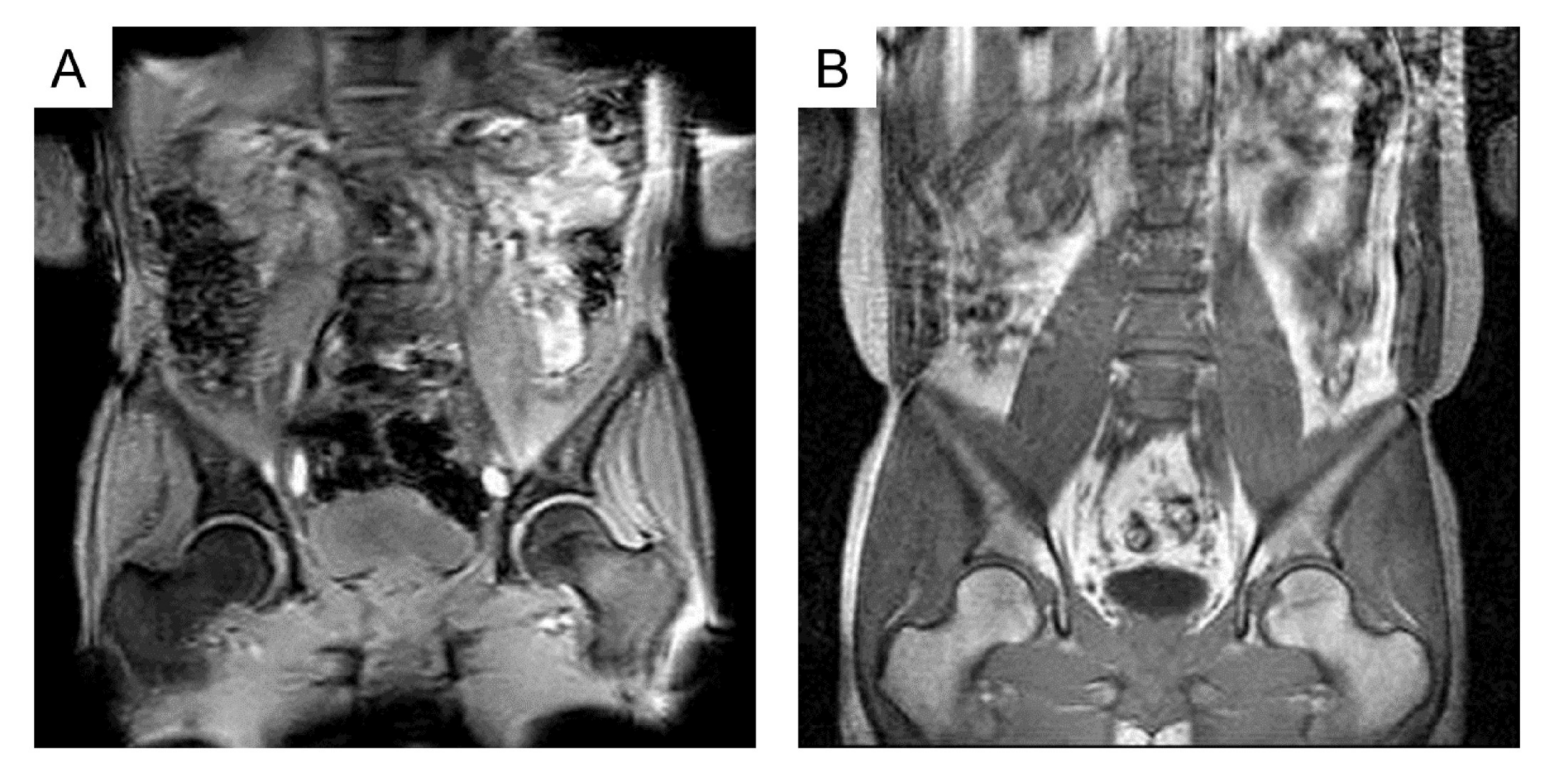
Comparison of scout images on 3 T gradient T2*-weighted image versus 1.5 T gradient proton density- weighted image. The signal drop of the bony structures and image distortion of the body edges are shown on the gradient T2*-weighted image (A) because of susceptibility effects, while both femoral heads are clearly depicted on the gradient proton density-weighted image (B).

When comparing the readability levels of scout images according to the MRI sequence in hospitals A and C, the percentages of readable cases for both femoral heads were significantly higher on 1.5 T GRE PDWI than on 3 T GRE T2*WI (hospital A; 83.3–90.0% vs. 16.7–20.0%, p<0.001, hospital C; 90.0–96.7% vs. 50.0–60.0%, p≤0.007), while that for both kidneys were significantly higher on 3 T GRE T2*WI than on 1.5 T GRE PDWI (hospital A; 83.3–100% vs. 33.3–66.7%, p≤0.006, hospital C; 70.0–86.7% vs. 20.0–43.3%, p≤0.001) by all readers (Table 3). The signal drop of the bony structures and image distortion of the body edge were observed on GRE T2*WI owing to susceptibility effects (Fig 3). When comparing the readability levels of scout images according to the number of coronal image slices on 3 T GRE T2*WI with the same slice thickness and intersection gap, the percentages of readable cases for both femoral heads (50.0–60.0% vs. 16.7–20.0%, p≤0.015) and necks (60.0–86.7% vs. 13.3–33.3%, p≤0.008) were significantly higher in hospital C (seven coronal plane slices) than in hospital A (three coronal plane slices) (Table 4). When comparing the readability levels of scout images according to the number and intersection gap of coronal image slices between hospital A (three coronal plane slices with 18-mm intersection gap) and hospital C (seven coronal plane slices with 1-mm intersection gap) on 1.5 T GRE PDWI with same slice thickness, the percentages of readable cases for both femoral necks were significantly higher in hospital C (86.7–93.3%) than in hospital A (40.0–50.0%) (p<0.001, Table 5).

**Table 3:** Comparison of the readability of scout images between 3 T gradient T2*-weighted image and 1.5 T gradient proton density-weighted image.

		Hospital A (n = 60)			Hospital C (n = 60)		
		3 T GRE T2*WI (n = 30)	1.5 T GRE PDWI (n = 30)		3 T GRE T2*WI (n = 30)	1.5 T GRE PDWI (n = 30)	
		Readable case number (%)		*P* value	Readable case number (%)		*P* value
Femoral head (Rt)	R1	5 (16.7)	26 (86.7)	<0.001^*a*^	15 (50.0)	27 (90.0)	0.002^*a*^
	R2	5 (16.7)	25 (83.3)	<0.001^*a*^	17 (56.7)	27 (90.0)	0.007^*a*^
Femoral head (Lt)	R1	6 (20.0)	27 (90.0)	<0.001^*a*^	18 (60.0)	29 (96.7)	0.001^*a*^
	R2	6 (20.0)	26 (86.7)	<0.001^*a*^	16 (53.3)	29 (96.7)	<0.001^*a*^
Femoral neck (Rt)	R1	4 (13.3)	12 (40.0)	0.039^*a*^	20 (66.7)	26 (86.7)	0.125
	R2	10 (33.3)	13 (43.3)	0.426	25 (83.3)	27 (90.0)	0.706
Femoral neck (Lt)	R1	7 (23.3)	12 (40.0)	0.165	18 (60.0)	28 (93.3)	0.005^*a*^
	R2	10 (33.3)	15 (50.0)	0.190	26 (86.7)	28 (93.3)	0.671
Sacroiliac joint (Rt)	R1	27 (90.0)	23 (76.7)	0.299	22 (73.3)	26 (86.7)	0.333
	R2	25 (83.3)	19 (63.3)	0.080	27 (90.0)	24 (80.0)	0.472
Sacroiliac joint (Lt)	R1	28 (93.3)	25 (83.3)	0.424	23 (78.3)	24 (80.0)	0.754
	R2	26 (86.7)	22 (73.3)	0.333	28 (93.3)	26 (86.7)	0.671
Kidney (Rt)	R1	30 (100.0)	18 (60.0)	<0.001^*a*^	25 (83.3)	10 (33.3)	<0.001^*a*^
	R2	25 (83.3)	10 (33.3)	<0.001^*a*^	21 (70.0)	6 (20.0)	<0.001^*a*^
Kidney (Lt)	R1	29 (96.7)	20 (66.7)	0.006^*a*^	26 (86.7)	13 (43.3)	0.001^*a*^
	R2	28 (93.3)	10 (33.3)	<0.001^*a*^	25 (83.3)	7 (23.3)	<0.001^*a*^

Readable case, definitely and possibly evaluable case; GRE T2*WI, gradient T2*- weighted image; GRE PDWI, gradient proton density-weighted image; R, reader.

^*a*^*p*<0.05

**Table 4:** Comparison of the readability of scout images according to the number of coronal image slices on 3 T gradient T2*-weighted image.

		Hospital A (n = 30)	Hospital C (n = 30)	*P* value
		Readable case number (%)		
Femoral head (Rt)	R1	5 (16.7)	15 (50.0)	0.013^*a*^
	R2	5 (16.7)	17 (56.7)	0.003^*a*^
Femoral head (Lt)	R1	6 (20.0)	18 (60.0)	0.003^*a*^
	R2	6 (20.0)	16 (53.3)	0.015^*a*^
Femoral neck (Rt)	R1	4 (13.3)	20 (66.7)	<0.001^*a*^
	R2	10 (33.3)	25 (83.3)	<0.001^*a*^
Femoral neck (Lt)	R1	7 (23.3)	18 (60.0)	0.008^*a*^
	R2	10 (33.3)	26 (86.7)	<0.001^*a*^
Sacroiliac joint (Rt)	R1	27 (90.0)	22 (73.3)	0.095
	R2	25 (83.3)	27 (90.0)	0.448
Sacroiliac joint (Lt)	R1	28 (93.3)	23 (76.7)	0.071
	R2	26 (86.7)	28 (93.3)	0.389
Kidney (Rt)	R1	30 (100.0)	25 (83.3)	0.020^*a*^
	R2	25 (83.3)	21 (70.0)	0.360
Kidney (Lt)	R1	29 (96.7)	26 (86.7)	0.161
	R2	28 (93.3)	25 (83.3)	0.228

Readable case, definitely and possibly evaluable case; R, reader

^*a*^*p*<0.05

**Table 5:** Comparison of the readability of scout images according to the number and intersection gap of the coronal image slices on 1.5 T gradient proton density-weighted image.

		Hospital A (n = 30)	Hospital C (n = 30)	*P* value
		Readable case number (%)		
Femoral head (Rt)	R1	26 (86.7)	27 (90.0)	1.000
	R2	25 (83.3)	27 (90.0)	0.706
Femoral head (Lt)	R1	27 (90.0)	29 (96.7)	0.612
	R2	26 (86.7)	29 (96.7)	0.353
Femoral neck (Rt)	R1	12 (40.0)	26 (86.7)	<0.001^*a*^
	R2	13 (43.3)	27 (90.0)	<0.001^*a*^
Femoral neck (Lt)	R1	12 (40.0)	28 (93.3)	<0.001^*a*^
	R2	15 (50.0)	28 (93.3)	<0.001^*a*^
Sacroiliac joint (Rt)	R1	23 (76.7)	26 (86.7)	0.506
	R2	19 (63.3)	24 (80.0)	0.252
Sacroiliac joint (Lt)	R1	25 (83.3)	24 (80.0)	1.000
	R2	22 (73.3)	26 (86.7)	0.333
Kidney (Rt)	R1	18 (60.0)	10 (33.3)	0.069
	R2	10 (33.3)	6 (20.0)	0.382
Kidney (Lt)	R1	10 (33.3)	13 (43.3)	0.119
	R2	10 (33.3)	7 (23.3)	0.567

Readable case, definitely and possibly evaluable case; R, reader

^*a*^*p*<0.05

## Discussion

Scout images are an integral part of MRI for appropriate localization of regions of interest. Medico-legal and ethical debates continue as to whether scout images should be a standard part of imaging interpretation due to limited image quality [1, 3–17, 19–21].

The present study was the first to assess the readability of scout images for evaluating extraspinal organs in lumbar spine MRI. In our study, more than 50% of femoral head, sacroiliac joint, and kidney cases were classified as readable cases. Femoral heads showed excellent interobserver agreements. With adequate protocols, more than 70% of femoral head cases were designated as readable cases. Different readability levels of scout images were observed according to the image protocols including the MRI sequence, number of coronal plane slices, and intersection gap of coronal plane slices.

Some previous studies investigated the readability and reliability of scout images for evaluation of vertebral fracture on lumbar spine MRI [22, 23]. Bazzocchi et al reported 100% sensitivity and specificity in the detection of vertebral fractures on scout images, with excellent inter-observer agreement and perfect intra-observer agreement [22]. Kaniewska et al. also documented that a whole spine scout image was a good diagnostic tool for the detection and evaluation of unsuspected vertebral fractures, with a strong inter-observer agreement [23]. These studies, unlike ours, analyzed the spine on scout images; however, we evaluated extraspinal organs that are often located at the edge of the scout images and may only be partially included in the images. Therefore, these studies cannot be directly compared to our study. However, both previous studies and our study showed that scout images in lumbar spine MRI are more than a mere technical component; they are readable and potentially contain additional clinical information on spinal and extraspinal organs.

Several previous studies documented incidental extraspinal findings on lumbar spine MRI including scout images, even though they did not distinguish the prevalence of incidental extraspinal findings on between the uncropped routine sequences and scout images [1, 3, 12, 13, 16]. Wagner et al. [1] noted that four cases of occult malignancies and one case of occult metastatic disease detected on coronal scout images. Kamath et al. [3] and Raghavan et al. [13] mentioned that the incidental findings on scout image of MRI may contain a wealth of information and it is important to incorporate evaluation of these images into routine practice in those review articles. However, the majority of incidental extraspinal findings of lumbar spine MRI were benign and asymptomatic. Reporting these insignificant incidental finding may lead to unnecessary further examinations or patient’s anxiety but is not related to lumbar symptom. Therefore, we concentrated on the exclusive diagnosis of clinically meaningful diseases in extraspinal organs on scout images as possible candidates for vague lumbar symptoms, and not incidental findings which are unintentionally encountered and not related to the patient’s symptoms. This is because if such clinically meaningful diseases are overlooked, the medical management time would be delayed, and medical malpractice litigation would be created.

Avascular necrosis of the femoral head and femoral neck fracture are common diseases that may mimic spinal disorders due to overlapping symptoms. Our study demonstrated that although avascular necrosis may be ruled out in the femoral head, the evaluation of femoral neck on scout images was limited due to incomplete coverage of this relatively small structure. A case of early-stage avascular necrosis was incidentally detected in our study, with a prevalence of 0.67% (1/150). Since some scout images did not include the femoral head or could not be evaluated due to artifacts or limited image quality, the prevalence was higher (1.19–1.28%) when the prevalence was calculated in the readable cases of the femoral head. Ibarhim et al. [24] noted that avascular necrosis of femoral head and acetabular fracture were incidentally found in 7 out of 400 patients who underwent lumbar spine MRI, although they were found in routine coronal sequences not scout images. The incidence of incidental avascular necrosis of the femoral head in lumbar spine MRI is not uncommon. We conjectured that the femoral head is the most consistently assessable organ in adequate scout image protocols of lumbar spine MRI. Thus, we encourage a careful survey of femoral head on scout image, especially when lumbar spine MRI failed to explain the symptom of the patient and no other image exist to screen the femoral head.

Unlike bone diseases, the ability of scout images to screen diseases involving the sacroiliac joint and kidneys was limited. Thin-section, fat saturation, T2-weighted images are necessary for investigating synovial joints such as the sacroiliac joint [25, 26]. Moreover, kidneys are frequently affected by motion artifacts or susceptibility artifacts on scout images. In our study, interobserver agreement for the evaluation of the sacroiliac joint and kidneys was not satisfactory.

Default MRI sequence, parameters, and image compositions of scout images in lumbar spine MRI may vary depending on MR vendors and institutions because no standard protocol exists. Wagner et al. [1] obtained scout images with gradient T1-weighted images, whereas Kamath et al. [3] and Bazzocchi et al. [22] used gradient T2-weighted images to identify incidental findings on lumbar spine MRI. Bazzocchi et al. [22] also showed examples of the different quality of scout images by modification of MRI acquisition techniques, however they did not directly compare the readability of scout images according to MRI acquisition techniques. Our study is the first to compare the readability of scout images according to different image protocols in lumbar spine MRI. In our study, readability level for evaluation of bony structures was better on GRE PDWI than on GRE T2*WI. Bone produces dark signal intensity which makes it difficult to decipher avascular necrosis or fracture lines on GRE T2*WI because of susceptibility effects. Moreover, the femoral head and neck were sometimes obscured by image distortion of the body edge. In our study, the number and intersection gap of coronal slices were important factors in determining the readability of scout images. Routine sequences (sagittal and axial T2- and T1- weighted images) of lumbar spine MRI do not usually include coronal scans. Therefore, a scout image is a unique image with coronal slice information. In our study, the femoral head and neck were only included in coronal scout images and the outlines of kidneys and iliac bone were more easily identified in these images. Our results demonstrated that as the slice number of coronal planes increased and the intersection gap of coronal planes decreased, the readability level for the evaluation of small and thin structures such as the femoral neck increased. Although there are limitations in establishing an optimal image protocol with this retrospective study, scout images in lumbar spine MRI are recommended to be obtained using GRE PDWI, not GRE T2*WI, with the appropriate number and intersection gap of coronal image slices for screening the femoral head and neck. Of course, the acquisition time of scout images is also a major variable. The acquisition time of scout image varied in our study from 16 s to 60 s depending on the protocols. However, it should not be overlooked that minor protocol changes and investing a few more seconds in obtaining scout images may help obtain additional information about extraspinal organs. Therefore, further image optimization is required for establishing standard scout image protocols considering the image quality, image acquisition time, and necessity of additional information of extraspinal organs according to clinical settings.

Our study has several limitations. First, imaging analysis was based on subjective interpretation by experienced radiologists. Second, the study sample size was small. Third, this study did not include pathological or clinical correlation. However, the primary goal of the study was to evaluate the readability of scout image according to the different protocols, not to determine the prevalence of incidental extraspinal findings in scout image and follow their outcome. Further prospective studies are needed to assess the true prevalence of various diseases in extraspinal organs covered only on scout images, to validate the diagnostic value of scout images. However, to our knowledge, this study represents the first study to investigate the readability of scout images for extraspinal organs in lumbar spine MRI. Our findings were compared to multicenter data using different scout image protocols to determine the protocol adequacy.

## Conclusion

In conclusion, scout images of lumbar spine MRI may be readable enough to rule out some major diseases of extraspinal organs. Standardization of the protocol will be needed to validate the potential role of scout images for screening extraspinal organs in lumbar spine MRI.
